# Breast Cancer-Specific Mortality Pattern and Its Changing Feature According to Estrogen Receptor Status in Two Time Periods

**DOI:** 10.1371/journal.pone.0157322

**Published:** 2016-06-14

**Authors:** Junjie Li, Yirong Liu, Yizhou Jiang, Zhimin Shao

**Affiliations:** 1 Department of Breast Surgery, Fudan University Shanghai Cancer Center, Fudan University, Shanghai, People’s Republic of China; 2 Department of Oncology, Shanghai Medical College, Fudan University, Shanghai, People’s Republic of China; National Institute of technology Rourkela, INDIA

## Abstract

**Purpose:**

To determine whether and how the patterns of breast cancer-specific mortality (BCSM) changed along with time periods.

**Methods:**

We used the Surveillance, Epidemiology and End Results registry to identify 228209 female patients diagnosed with invasive breast cancer between 1990 and 2000 (cohort 1 [C1], 112981) and between 2001 and 2005 (cohort 2 [C2], 115228). BCSM was compared in two cohorts using Cox proportional hazard regression models. We analysed the relative hazard ratios (HRs) and absolute BCSM rates by flexible parametric survival modelling.

**Results:**

The patterns of BCSM were similar between the two cohorts, with the peak of mortality presenting in the first 2–3 years after diagnosis, and mortality rate significantly decreased in C2 in all cases. In C2, the annual BCSM rate of all cases was 9.64 (per 1000 persons per year) in year 10 with a peak rate of 23.34 in year 2. In ER-negative and high-risk patients, marked survival improvements were achieved mostly in the first 5 years, while in ER-positive and low-risk patients, survival improvements were less but constant up to 10 years.

**Conclusion:**

There has been a significant improvement of BCSM with substantially decreased mortality within 5 years. The current pattern of BCSM and its changing feature differs according to ER status. Our findings have some clinical implications both for treatment decisions and adjuvant treatment trial design.

## Introduction

Breast cancer is the most frequently occurring cancer among women worldwide. In 2012, 1.67 million new cancer cases (approximately one in four of all cancers among women) and 0.52 million cancer-related deaths were reported, with an estimated 5-year prevalence of 6.23 million.[1]Through a combination of early detection and more effective treatments, the mortality rate was reported to have decreased over the last three decades in most Western countries, 5-year net survival for women diagnosed with breast cancer had increased in many regions and countries recently, for example over 85% in Canada and US.[2, 3]

Breast cancer is now classified according to molecular factors that predict response to treatment, such as endocrine therapy to Luminal diseases, trastuzumab to HER2 positive diseases and chemotherapy to triple negative diseases, [4]and each intrinsic subtype has a unique risk of recurrence and death overtime.[5]The particular patterns of relapse and death differed according to these factors, and these patterns notably persisted with current therapies and improved over the last decades. In a large retrospective analysis, two cohorts of patients with breast cancer treated during two separate time periods were compared; outcomes improved for patients with all breast cancer subtypes, especially HER2-positive and ER-negative/HER2-negative cancers, with a marked decrease in the early spike in disease recurrence.[6]Due to the survival improvements, in many clinical trials, even global multicenter trials, the exact recurrence and/or mortality risks of the study population were much lower than the estimated risk when the trials were designed, led to extend follow-up time, to adjust study end points, or unable to achieve statistical power. Hence, the timing and patterns of breast cancer-specific mortality (BCSM) is important for treatment decisions, patient discussions, and designing clinical trials. Whether and how the patterns of 10 years BCSM (in the total population or certain subtypes) changed has not been studied in population-based database. In the present analysis, our aim was to demonstrate the current patterns of BCSM in patients treated in the modern treatment era (2001 to 2005) compared with a historic cohort from 1990 to 2000.

## Materials and Methods

### Patient selection and Outcome measures

To collect sufficient cases, we used the National Cancer Institute’s Surveillance, Epidemiology, and End Results (SEER) cancer database.[7] The current SEER database consists of 18 population-based cancer registries. We selected female patients with invasive breast cancer between January 1, 1990, and December 31, 2005. Eligible patients were divided into two cohorts according to different time periods: cohort 1 (C1) between January 1, 1990and December 31, 2000, and cohort 2 (C2) between January 1, 2001 and December 31, 2005. Patients diagnosed before 1990 were excluded due to unavailable hormone receptor data; patients diagnosed after 2005 were excluded to ensure adequate follow-up time.

**Fig 1:**
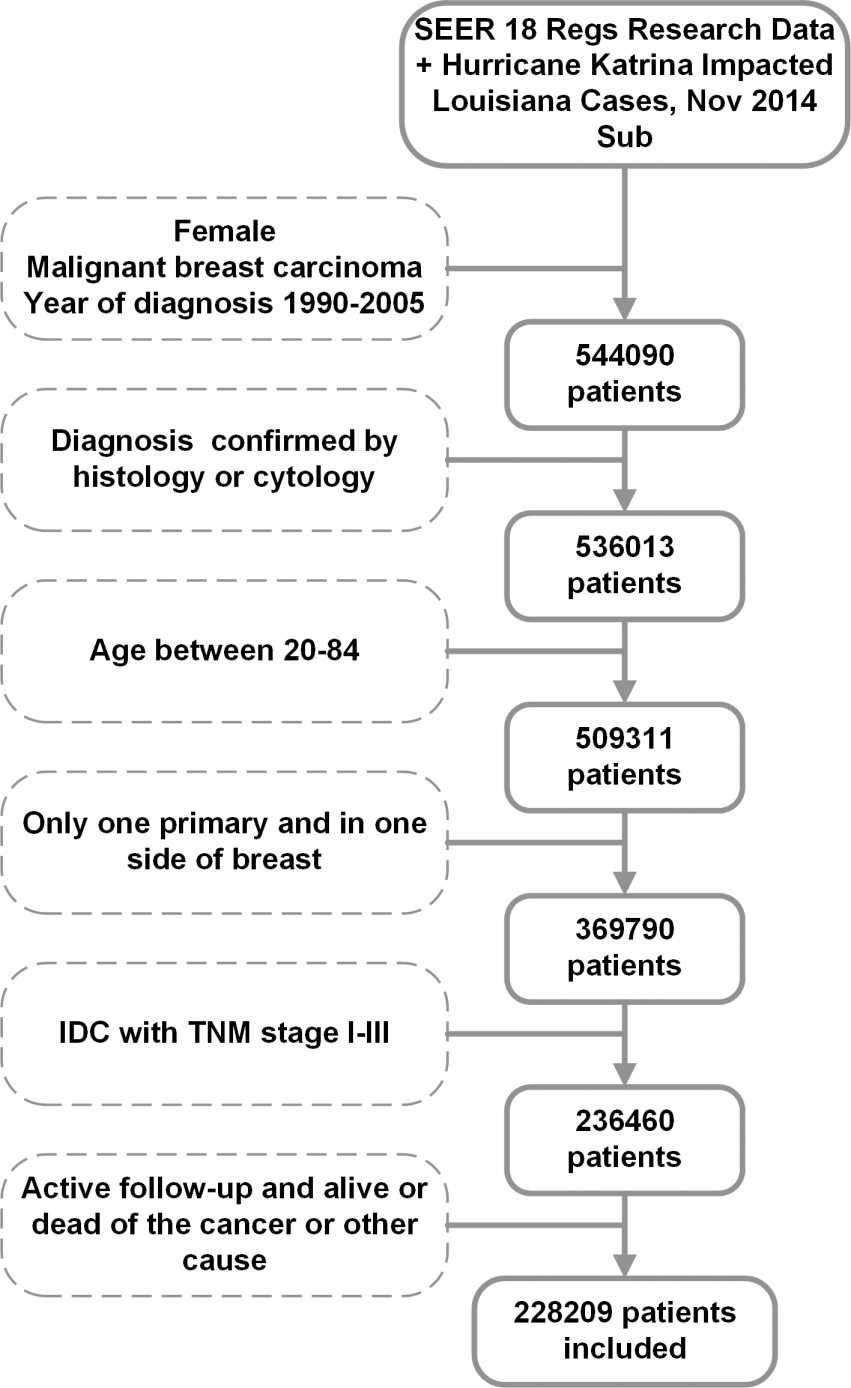
Filtration process. We identified 228209 female patients (20 and 84 yrs) diagnosed stages I to III unilateral invasive breast cancer between January 1, 1990, and December 31, 2005, through Surveillance, Epidemiology, and End Results (SEER) Program (www.seer.cancer.gov) SEER*Stat Database: Incidence—SEER 18 Regs Research Data released April 2015, based on the November 2014 submission.

We identified 228209 patients in the SEER database according to the following inclusion criteria: female, pathologically confirmed invasive ductal carcinoma (IDC, ICD-O-3 8500/3), age at diagnosis between 20 and 84 yrs, surgical treatment with either mastectomy or breast-conserving surgery, American Joint Committee on Cancer (AJCC) stages I to III, unilateral breast cancer, known ER status, known time of diagnosis, and breast cancer as the first and only cancer diagnosis (Fig 1). Information on the following variables was obtained if available: tumour size, histological grade, race, marital, and use or not use of radiotherapy. Because SEER does not provide information on chemotherapy and endocrine therapy, we could not incorporate and adjust for these variables. For this study, BCSM, which was the primary study outcome, was calculated from the date of diagnosis to the date of death caused by breast cancer. Patients who died of other causes were censored on the date of death.

This research was submitted to the Ethical Committee and Institutional Review Board at the Shanghai Cancer Centre of Fudan University and determined to be qualified for institutional review board exemption. The data released through the SEER database do not require informed patient consent because cancer is a reportable disease in every state in the US.

### Data management and statistical analysis

Age was categorized into <40, 40–60, and ≥60 years groups. Race and ethnicity were coded as white, black, and other (American Indian/AK Native, Asian/ Pacific Islander). Marital status was coded as married and not married (including divorced, widowed, single (never married) and separated). Tumour characteristics included tumour size, histological grade, lymph nodes status, ER status, PR status, and radiotherapy.

All analyses in the present study were conducted in a 10-yr frame to guarantee the validity and reliability of the results. Cox proportional hazard regression models were applied to estimate hazard ratio in different subgroups.[8–10]We hypothesised that the effect of prognostic factors on survival were changing with time. Thus when we calculated a time-dependent effect, the flexible parametric survival models were used to model the outcomes which allows covariates to have time-dependent effects by using spline function. BCSM, difference in mortality rate and hazard ratio were estimated using default parameters setting in the flexible models.[11]The baseline rates were estimated using a spline with five degrees of freedom, as previously stated.[10] Using spline function, we could study whether the effect of covariates on survival were dependent on follow-up time. In the present study, all factors were treated as constant in multivariate regression analysis. The flexible parametric survival model was analysed using the stpm2 packages in Stata (StataCorp, College Station, TX, Version 12). A two-sided P lower than 0.05 indicated statistical significance.

## Results

The SEER database identified 228209 eligible patients for the analysis, with 112981 patients in C1 and 115228 patients in C2. Table 1 summarized basic characteristics of the study patients. No significant difference was observed between the two cohorts with respect to basic characteristics. Median follow-up times were 153 (89–193) and 102 (86–122) months in C1 and C2, respectively.

**Table 1:** Patient characteristics.

Characteristics	Total*n*.228209	Year of Diagnosis	
		1990–2000*n*. (%)	2001–2005*n*. (%)
		112981	115228
**Median follow-up (IQR, months)**	116 (86–153)	153 (89–193)	102 (86–122)
**Age**			
**<40**	17540	9072(8.0)	8468(7.3)
**40–59**	108285	51067(45.2)	57218(49.7)
**≥60**	102384	52842(46.8)	49542(43.0)
**Marital status**			
**Married**	135559	66507(58.9)	69052(59.9)
**Not married**	86363	43043(38.1)	43320(37.6)
**Unknown**	6287	3431(3.0)	2856(2.5)
**Grade**			
**I**	34481	14927(13.2)	19554(17)
**II**	86295	41667(36.9)	44628(38.7)
**III**	90416	43183(38.2)	47233(41.0)
**Unknown**	17017	13204(11.7)	3813(3.3)
**T stage**			
**T1**	146525	72923(64.5)	73602(63.9)
**T2**	67343	33306(29.5)	34037(29.5)
**T3**	8867	4257(3.8)	4610(4.0)
**T4**	5474	2495(2.2)	2979(2.6)
**N stage**			
**N0**	148632	74147(65.6)	74485(64.6)
**N1**	53453	24919(22.1)	28534(24.8)
**N2**	17236	9058(8.0)	8178(7.1)
**N3**	8888	4857(4.3)	4031(3.5)
**ER**			
**Positive**	145813	69726(61.7)	76087(66.0)
**Negative**	51010	24358(21.6)	26652(23.1)
**Borderline**	1067	807(0.7)	260(0.2)
**Unknown**	30319	18090(16.0)	12229(10.6)
**PR**			
**Positive**	123544	59813(52.9)	63731(55.3)
**Negative**	68772	31751(28.1)	37021(32.1)
**Borderline**	1793	978(0.9)	815(0.7)
**Unknown**	34100	20439(18.1)	13661(11.9)
**Radiation**			
**Yes**	114107	52356(46.3)	61751(53.6)
**No**	114102	60625(53.7)	53477(46.4)

Abbreviations: ER, estrogen receptor; IQR, Interquartile range; PR, progesterone receptor.

### Pattern of breast cancer-specific mortality

**Fig 2:**
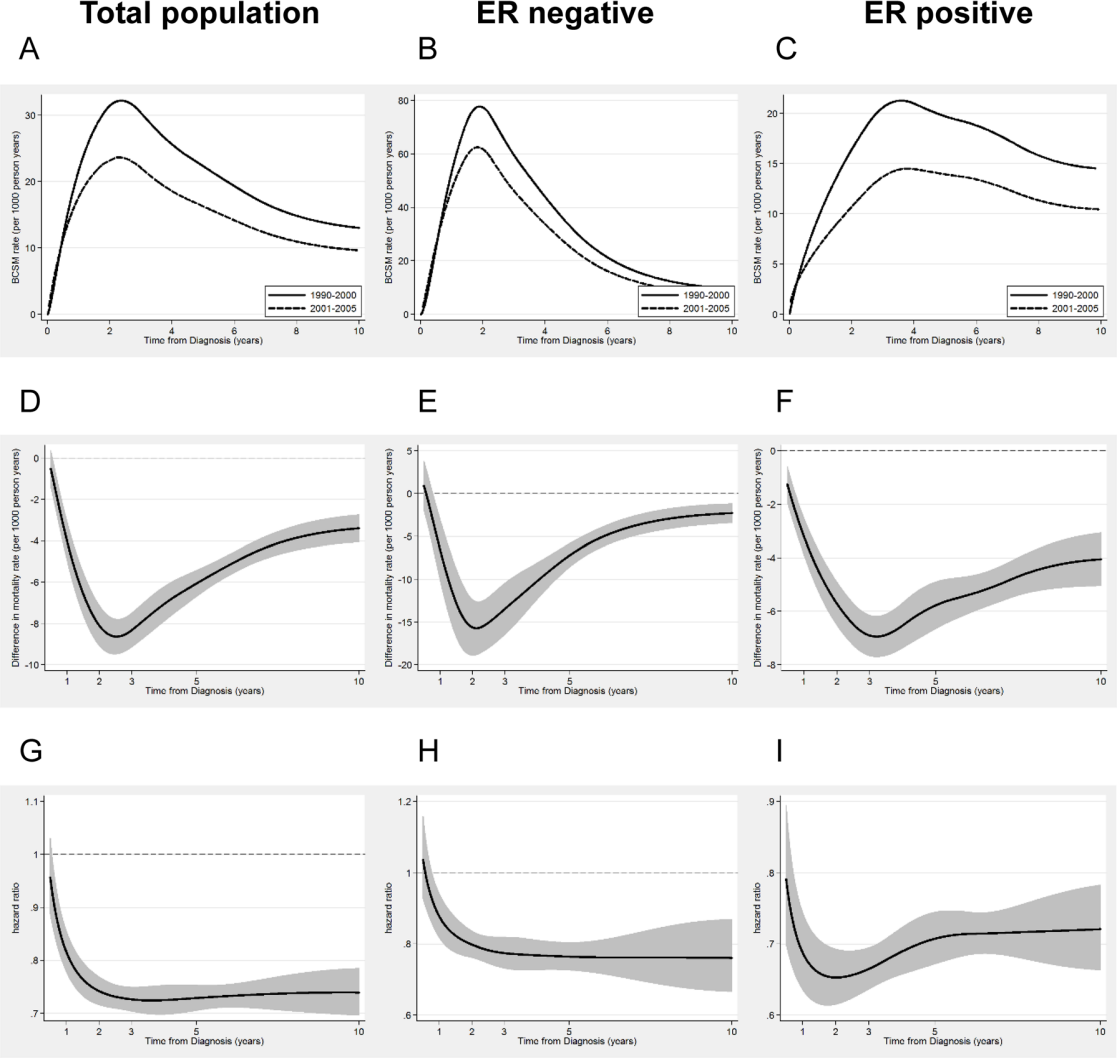
Estimated continuous annual BCSM rates and hazard ratio of BCSM. A, B & C show estimated continuous annual BCSM rates. The solid line represents patients in cohort 1; the dashed line represents patients in cohort 2.D, E & F show differences of BCSM between patients in the two cohorts. Patients in C1 as reference, the absolute number of decreased BCSM rate (C2 minus C1).G, H & I show hazard ratio of BCSM (Cohort 1 versus cohort 2). Light grey shadows represent 95% CI of survival. HRs with 95% CIs were estimated from the flexible parametric survival models. Curves cut off at 0.5 yrs from diagnosis because of sparse data. Total population: A, D & G; ER negative: B, E & H; ER positive: C, F & I. Notes: Rates were reported per 1000 persons per year; y-axis scales were different in different analyses.

Fig 2A, 2B and 2C showed estimated continuous annual BCSM rates in the total population, ER negative and ER positive subgroups. BCSM rate was reported per 1000 persons per year. The patterns of BCSM were similar between the two cohorts, and the hazard curves for BCSM both peaked at 3 years after initial diagnosis. The risk of breast cancer deaths was nonproportional overall and differed by estrogen receptor (ER) status. In ER-negative patients, the peak shifted to an earlier time (year 2), with an annual mortality rate of61.83 (per 1000 persons per year). Then, the curve declined sharply, with an annual mortality rate of less than 20 after the fifth year. The annual hazard in ER-positive patients accumulated through the first 4 years and reached a peak at the fourth year after diagnosis. After four years, the hazard rate plateaued and remained stable for a long time. For example, the annual BCSM rates were maintained at 10–15 from year 2 to year 10 in C2. Patients with a node-positive, larger tumour size (T3) or younger age (<40 y) showed an early major mortality surge, peaking at year 2, which was similar to ER-negative cancers. Patients with node-negative, smaller tumour size (T1) or older age (≥60 y) had no sharp peaks and a lower annual BCSM rates similar to ER-positive tumours. Remarkably, long-term mortality risks remained in C2.The annual BCSM rate of all cases was 9.64 in year 10 and was approximately 20 in year 10 for aggressive patients (such as node-positive, T3, or <40 y).(Data listed in Table 2)

**Table 2:** Annual BCSM rates and hazard ratio of BCSM in the total population and different subgroups.

	BCSM rate (per 1000 person year)									
**All**	0–1 y	1–2 y	2–3 y	3–4 y	4–5 y	5–6 y	6–7 y	7–8 y	8–9 y	9–10 y
**C1**	21.68	31.45	30.39	25.61	22.32	19.33	16.67	14.84	13.7	13.03
**C2**	17.67	23.34	22.07	18.57	16.27	14.17	12.27	10.96	10.13	9.64
**HR C2/C1**	0.815	0.742	0.726	0.725	0.729	0.733	0.736	0.738	0.739	0.739
**95%CI**	0.778–0.854	0.716–0.769	0.704–0.749	0.698–0.753	0.706–0.753	0.712–0.755	0.709–0.764	0.704–0.774	0.700–0.781	0.697–0.785
**ER-**										
**C1**	53.46	77.46	59.53	43.91	30.48	21.29	15.73	12.44	10.56	9.55
**C2**	46.89	61.83	46.09	33.72	23.30	16.23	11.98	9.48	8.04	7.27
**HR C2/C1**	0.877	0.798	0.774	0.768	0.764	0.762	0.762	0.762	0.762	0.761
**95%CI**	0.818–0.941	0.762–0.836	0.732–0.819	0.728–0.811	0.727–0.804	0.717–0.811	0.7–0.829	0.684–0.849	0.672–0.863	0.666–0.869
**ER+**										
**C1**	10.21	16.44	20.61	20.98	19.72	18.81	17.39	15.82	14.90	14.51
**C2**	7.02	10.73	13.70	14.47	13.95	13.43	12.44	11.35	10.71	10.46
**HR C2/C1**	0.688	0.653	0.665	0.689	0.707	0.714	0.715	0.717	0.719	0.721
**95%CI**	0.636–0.744	0.615–0.693	0.637–0.695	0.657–0.723	0.672–0.744	0.685–0.745	0.685–0.747	0.676–0.761	0.668–0.774	0.664–0.783
**LN-**										
**C1**	8.29	13.72	15.87	13.98	12.41	11.26	10.13	9.24	8.70	8.41
**C2**	7.66	11.27	12.27	10.33	8.95	8.02	7.13	6.44	6.01	5.76
**HR C2/C1**	0.925	0.821	0.774	0.739	0.721	0.712	0.704	0.697	0.691	0.685
**95%CI**	0.852–1.005	0.771–0.875	0.736–0.813	0.695–0.785	0.679–0.765	0.678–0.747	0.665–0.745	0.646–0.751	0.632–0.755	0.621–0.756
**LN+**										
**C1**	48.11	67.61	61.62	51.89	45.30	38.60	32.73	28.86	26.44	25.04
**C2**	36.27	46.43	41.74	35.48	31.48	27.24	23.41	20.86	19.24	18.28
**HR C2/C1**	0.754	0.687	0.677	0.684	0.695	0.706	0.715	0.723	0.728	0.730
**95%CI**	0.712–0.798	0.658–0.717	0.65–0.705	0.652–0.717	0.669–0.722	0.68–0.733	0.681–0.751	0.68–0.768	0.678–0.781	0.677–0.788
**T1**										
**C1**	7.32	12.52	15.27	14.14	12.61	11.82	10.98	10.20	9.73	9.49
**C2**	5.10	7.98	9.80	9.34	8.48	7.94	7.32	6.77	6.43	6.26
**HR C2/C1**	0.697	0.637	0.642	0.661	0.672	0.672	0.667	0.663	0.661	0.660
**95%CI**	0.635–0.765	0.594–0.684	0.608–0.676	0.622–0.701	0.631–0.717	0.638–0.707	0.631–0.704	0.617–0.713	0.605–0.722	0.598–0.728
**T2**										
**C1**	34.58	57.06	55.72	46.07	39.80	33.72	28.40	24.87	22.70	21.47
**C2**	27.70	40.81	38.98	32.56	28.82	25.04	21.57	19.24	17.77	16.92
**HR C2/C1**	0.801	0.715	0.700	0.707	0.724	0.743	0.760	0.774	0.783	0.788
**95%CI**	0.748–0.857	0.679–0.753	0.669–0.732	0.669–0.746	0.691–0.758	0.711–0.775	0.719–0.803	0.721–0.83	0.721–0.85	0.721–0.861
**T3**										
**C1**	86.85	117.82	89.62	76.12	61.86	47.94	38.70	32.88	29.32	27.26
**C2**	76.34	95.80	71.12	60.44	49.14	38.06	30.74	26.13	23.29	21.64
**HR C2/C1**	0.879	0.813	0.794	0.794	0.794	0.794	0.794	0.795	0.795	0.794
**95%CI**	0.769–1.005	0.741–0.892	0.709–0.889	0.713–0.884	0.725–0.871	0.712–0.886	0.687–0.918	0.665–0.95	0.648–0.974	0.639–0.986
**Age<40Y**										
**C1**	33.99	53.05	48.41	44.36	36.32	29.70	25.00	21.93	20.02	18.92
**C2**	22.94	35.78	33.51	31.70	27.00	23.00	20.06	18.09	16.85	16.13
**HR C2/C1**	0.675	0.675	0.692	0.715	0.744	0.774	0.802	0.825	0.842	0.852
**95%CI**	0.575–0.792	0.599–0.76	0.632–0.758	0.642–0.796	0.673–0.822	0.709–0.846	0.72–0.894	0.718–0.948	0.715–0.99	0.714–1.017
**40-59Y**									
**C1**	20.87	31.51	29.82	24.60	21.30	18.22	15.40	13.47	12.29	11.62
**C2**	16.03	21.99	20.76	17.37	15.19	13.04	11.04	9.69	8.85	8.37
**HR C2/C1**	0.768	0.698	0.696	0.706	0.713	0.715	0.717	0.719	0.720	0.720
**95%CI**	0.716–0.824	0.66–0.737	0.666–0.728	0.668–0.747	0.679–0.749	0.685–0.747	0.68–0.757	0.67–0.771	0.663–0.781	0.659–0.787
**Age**≥**60**										
**C1**	20.66	27.81	27.13	23.87	21.04	18.59	16.55	15.13	14.21	13.66
**C2**	19.09	23.00	21.09	17.99	15.77	13.98	12.47	11.42	10.71	10.27
**HR C2/C1**	0.924	0.827	0.777	0.754	0.749	0.752	0.754	0.754	0.754	0.752
**95%CI**	0.861–0.992	0.785–0.871	0.739–0.817	0.712–0.798	0.714–0.787	0.719–0.787	0.712–0.799	0.702–0.811	0.694–0.819	0.687–0.822
**Radiation**										
**C1**	17.28	26.72	25.46	22.80	19.92	17.15	14.64	12.89	11.82	11.23
**C2**	12.31	20.23	18.88	16.55	14.57	12.92	11.34	10.21	9.5	9.1
**HR C2/C1**	0.712	0.757	0.741	0.726	0.731	0.753	0.774	0.792	0.804	0.81
**95%CI**	0.657–0.773	0.713–0.804	0.708–0.777	0.686–0.767	0.693–0.771	0.721–0.787	0.734–0.816	0.739–0.848	0.741–0.872	0.741–0.886
**Non-Radiation**										
**C1**	26.17	35.63	33.68	28.88	24.95	21.47	18.61	16.68	15.46	14.72
**C2**	23.95	28.31	25.26	21.12	18.13	15.56	13.45	12.03	11.11	10.53
**HR C2/C1**	0.915	0.794	0.749	0.731	0.726	0.725	0.722	0.72	0.718	0.715
**95%CI**	0.861–0.972	0.76–0.83	0.717–0.783	0.695–0.768	0.696–0.757	0.696–0.755	0.686–0.761	0.675–0.769	0.666–0.774	0.659–0.775

Abbreviations: BCSM, breast cancer-specific mortality; ER, estrogen receptor; HR, hazard ratio; LN, lymph node.

### Comparison of BCSM between two cohorts

Despite the same pattern of BCSM over time between the two study cohorts, the annual BCSM rates of C2 decreased throughout the yearly intervals in all cases. The absolute number for decreased mortality rate (C2 minus C1) was used to demonstrate the differences in BCSM rate between two cohorts. Fig 2D, 2E and 2F showed that the survival difference curve formed a V-shaped curve, indicating that the majority of survival improvements in C2 were in the first 5 years after diagnosis. In the total population, the survival difference between the two cohorts continuously increased until a peak was reached2-3 years from diagnosis (absolute decreased BCSM rate was approximately 10), after which the survival difference lessened to under 5 after the fifth year. In ER-negative patients particularly, the peak annual BCSM rate was at year 2 when the improved absolute BCSM rate was 15.The curve then dropped rapidly, with an improved BCSM rate of less than 5 by the fifth through tenth years. In ER-positive patients, survival improvement differences were smaller between the two cohorts, with a steadily improved BCSM rate of 6 to 4 from the fifth year to the tenth year.

### Hazard ratio of BCSM in the total population and different subgroups

The HRs of BCSM between the two cohorts (C2 versus C1) provided further information. As shown in Fig 2G, 2H and 2I, the HRs of BCSM were steady over time, indicating that there were sustained survival improvements 10 years from diagnosis in C2. In patients with aggressive diseases, the curves of HRs were close to 1 in year 10, especially for those age <40yrswhose tenth year HR was 0.852 (95%CI:0.714–1.017)(Table 2).This datum indicated that survival improvements were greater in early years from diagnosis and were not sustained for 10 years.

### Survival improvement in subpopulations

**Fig 3:**
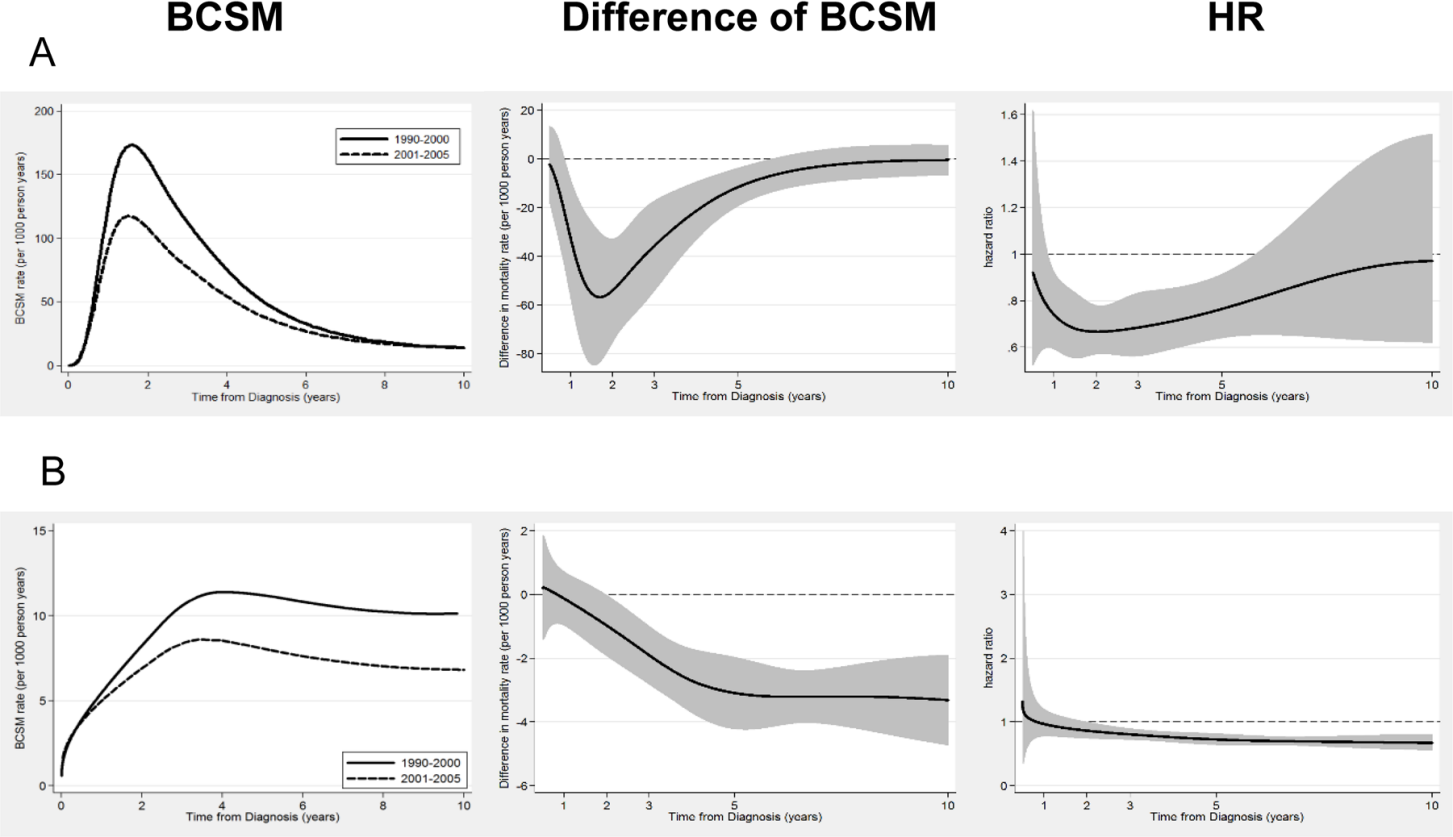
Estimated continuous annual BCSM rates and hazard ratio of BCSM in certain subpopulations. (A) Patients were age<40 with ER negative and node positive diseases. (B) Patients were age≥60 with ER positive and node negative diseases.

**Fig 4:**
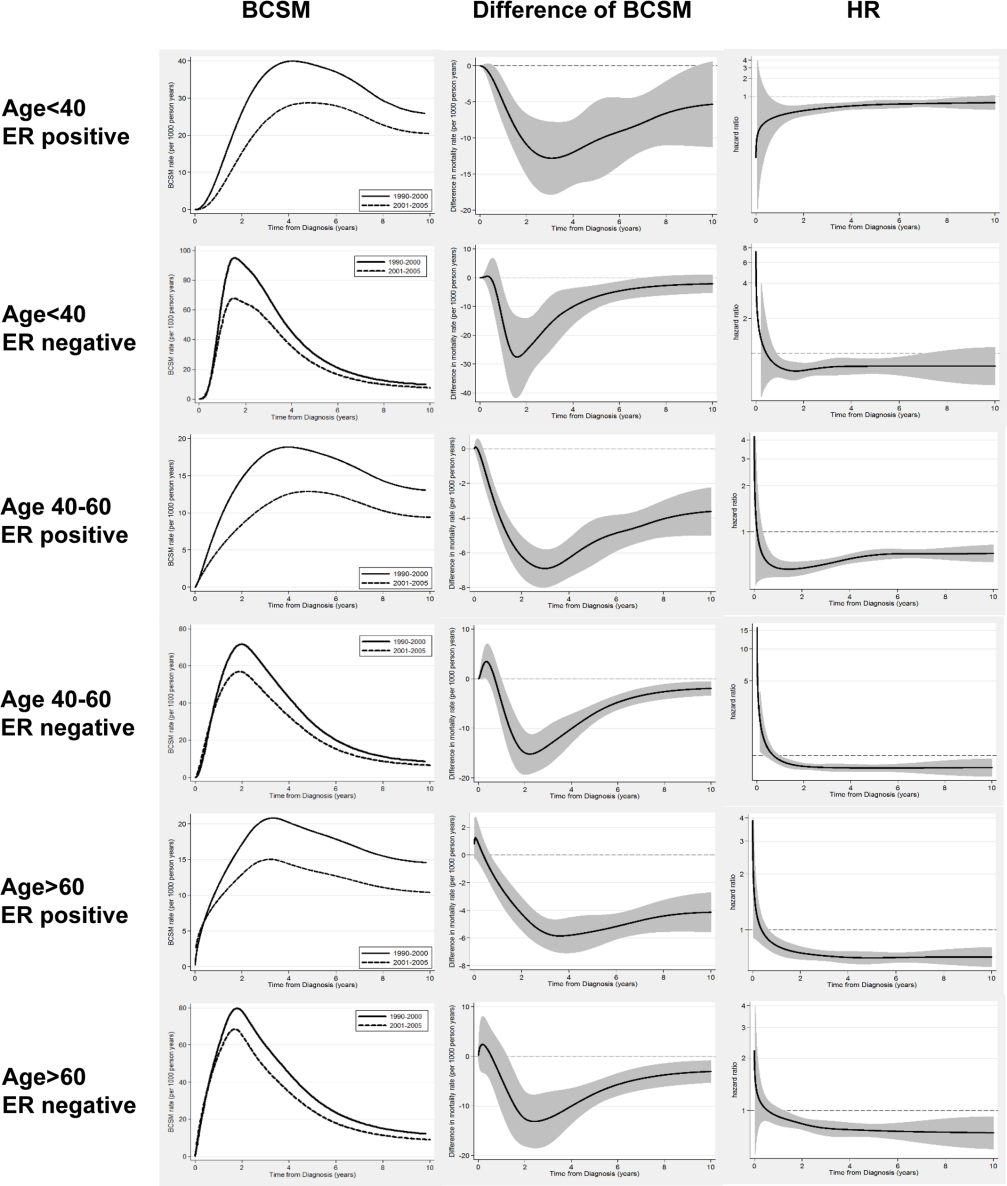
Estimated continuous annual BCSM rates and hazard ratio of BCSM according to age and ER status.

We further inspected whether survival improvements of C2 differ in subpopulations according to ER status, age at diagnosis and node status. We found that patients with aggressive tumours, such as those ER-negative, node-positive and age <40yrs, had a higher BCSM rate over time, and substantial survival benefit of C2 occurred in the first 5 years from diagnosis (peaking at the second year), with almost no survival benefit after the fifth year (Fig 3A). However, patients with low-risk diseases, such as those ER-positive, node-negative and age ≥60yrs, had a lower BCSM rate over time, and the survival benefit in C2 was smaller and was sustained to the tenth year (Fig 3B). Fig 4 showed how the patterns of BCSM changed according to ER status and different age groups.

## Discussion

We sought to determine whether and how the patterns of BCSM changed along with time periods in a large retrospective analysis using the SEER database. Our findings confirmed a reduction of BCSM in the C2 period and demonstrated changing feature of BCSM patterns differed according to ER status.

Usually, the decrease in mortality rates in Western countries were likely explained by the implementation of mammography screening and the use of more comprehensive and appropriate systemic and locoregional treatments.[3]Mammography screening has been implemented in the US since the 1980s. [12] Therefore, in the current study there was no lead time bias or length bias created by mammography screening [13]and clinicopathological characteristics (grade, T and N stage) of patients in the two cohorts were similar. Hence, we believe that the survival improvement in C2 was largely due to more effective treatment strategies, in agreement with recent study showed a key role for treatment of stage-specific survival improvement in US. [14]Although third-generation aromatase inhibitors and cytotoxic drugs, such as taxanes, have been approved by the Food and Drug Administration (FDA) to treat early breast cancer since 2005 and 2006, respectively,[15] there were more anthracycline-based adjuvant regimens (rather than CMF),more standard use of Tamoxifen, and an increasingly individualized treatment approach for metastatic disease in C2. [16, 17]

Our data demonstrated that the pattern of mortality remained similar in a recent cohort both in ER-positive and ER-negative patients, with the peaks of the curves in C2 becoming attenuated. The similar BCSM patterns demonstrated that the improvements in adjuvant treatments led to more cured patients rather than just postponed recurrences or death from the primary tumour.

We also found that the curves of annual BCSM rates peaked at 3 years after diagnosis, and aggressive diseases presented higher and earlier peaks. The pattern of BCSM differed according to ER status. A previous study has demonstrated the peak hazard of recurrence occurred in the interval of 1 to 2 years for the entire population[18], with the peak of recurrence for luminal A at 36 months postoperatively and the peak of recurrence for HER2-enriched and TNBC at 12 months.[5, 19]Furthermore, there is a close relationship between ER status and metastasis-specific survival. Patients with ER-negative/HER2-negative tumours had a median survival of 10 months after the detection of distant metastasis, and those with ER-negative/HER2-positive tumours had a median survival of 19 months. Patients with ER-positive tumours had a median survival time of 25 months (Her2-positive or -negative).[20] Our data were in agreement with published literature that stated that ER-negative tumours had earlier recurrence risks and shorter survival time after recurrence, and we demonstrated that the peak hazard of mortality was 4 years after diagnosis in ER-positive patients and 2 years in ER-negative patients.

Our study analyzed more eligible patients with a longer follow up in C2of approximately 102 months, our study was able to show the long term change of BCSM patterns and indicated that the survival benefit formed a V-shaped curve, with greater survival improvements in the first 5 years after diagnosis, especially in the ER-negative patients, which is consistent with and further complements the results of Cossetti et al. [6]The risk of breast cancer death (hazard rate) varies over time and is nonproportional, due to the nonproportional treatment effects of adjuvant therapy.[21] The biology of the disease, inferred by BC subtypes, appears to be the major determinant. For ER-negative patients, as shown in Fig 4, the survival benefit decreased substantially in all age groups after 5 years. One possible reason is that there was a higher annual mortality rate in the first 5 years in ER-negative patients, making it much easier to achieve a significant survival benefit during this time period through more effective treatments. Another possible reason is that for ER-negative patients, chemotherapy was the only treatment resulting in dramatic decreases in early recurrence rates, and the benefit from chemotherapy is known to occur mostly in the initial 3 years after treatment, with no rebound effect. [22]

For ER-positive patients in our study, the standard adjuvant endocrine therapy was Tamoxifen for 5 years, and some patients in C1 used Tamoxifen for 2 years before the results of the Swedish trial were available.[23]Different from chemotherapy, Tamoxifen has been reported to have a “carryover effect”, which is seen mostly in the first 5 years after treatment cessation.[24]Our data showed that the peak of the hazard rate for mortality almost vanished in ER-positive patients in C2, with a plateau of hazard rate emerging and remaining stable for a long time. The annual BCSM rates were maintained at 10–15 from year 2 to year 10, which was consistent with previous studies that reported that ER-positive tumours have low recurrence rates initially and a constant and unrelenting risk of relapse that extends up to 15 years.[25, 26]Annual BCSM rates were different according to age groups in ER-positive patients in C2, which were 10–15 (per 1000 persons per year) in 10 years in patients aged 40–59 and ≥60 years, and almost doubled in patients younger than 40 years (Fig 4). The outcome for ER-positive patients may change timely., After the results of ATALS and MA17 trials, [27, 28] more ER-positive patients, especially those with a high-risk disease, will receive extended adjuvant endocrine therapy and have further improved survival. The worse outcome in younger patients may also improve, since recently published results from the SOFT and TEXT trials indicate that more high risk younger patients may use ovarian function suppression therapy in adjuvant setting. [29, 30]On the other hand, the lowed BCSM rate in the total population questioned the necessity of using such aggressive treatments to the total population, and indicated the importance of searching new methods to identify the real high risk patients precisely. The improving prognosis emphasizes the importance of periodically revisiting the long-term hazard rate of BCSM patterns to guide treatment decisions.

Patterns of survival improvement differed in different subgroups in our analysis. For example, in the high-risk subgroup (ER-negative, node-positive and age<40 yrs, mostly only receiving chemotherapy) showed substantially decreased early mortality rates. However, the reduced but persistent peak of mortality still existed, the annual hazard rate peaked over 100 per persons per year in the second year after diagnosis and over 20 by the tenth year, signalling the need for new treatment strategies in future studies. Meanwhile, in the low-risk group (ER-positive, node-negative andage≥60 yrs, mostly receiving only endocrine therapy), the mortality rate was lower but persistent. Future studies may focus on how to use well-tolerated long-term endocrine therapy to abolish the mortality peak, keep the annual mortality rate less than 5 through the first 10 years, and cure these cancers eventually.

Our study had several limitations. First, we used ER status without HER2 status as the only marker to identify breast cancer subtypes. We used ER rather than ER and/or PR to define an endocrine sensitive tumour because ER status was thought to be the only recorded factor importantly predictive of the proportional reductions from endocrine therapy.[26]Knowledge of HER2 status would permit further classification of ER-positive tumours as luminal-A (HER2-negative) or luminal-B (HER2-positive). However, HER2 status was not available from the SEER database, so we failed to define the unique BCSM pattern of HER2-positive disease. On the other hand, in the two cohorts, adjuvant trastuzumab was not used nationwide in patients with Her2-positive diseases because adjuvant use of trastuzumab was approved in the US in 2006 [15].Thus, a lack of HER2 information may not affect the survival improvements of C2. Second, the SEER data lacks detailed information on recurrence events and cancer therapy (adjuvant systemic treatment). Therefore, we cannot validate the recurrence curves in US patients to demonstrate how the pattern of recurrence changed in the two cohorts throughout our analysis. However, the SEER database provided a large analysis population and long follow-up time, which is thought to be adequate in our analysis for identifying the changed pattern of BCSM in the total population and in certain subgroups, which were divided by ER status. Third, there were less than 1% patients in both cohorts with ER/PR borderline diseases. There are five codes for ER/PR status in SEER database: 1 positive, 2 negative, 3 r borderline, 4 unknown and 9 before 1990. The definition of borderline was "undetermined whether positive or negative", and we believed the low ratio of borderline diseases may not affect the results of our study.

In conclusion, our study first indicated that along with improved treatment, annual BCSM rates approximately 10–20 per 1000 persons per year10-yr frame in the modern era. In ER-negative and high-risk patients, marked survival improvement was achieved mostly in the first 5 years, while in ER-positive, survival improvement was less but constant up to 10 years. The updated patterns of BCSM and changing trends are of great importance to physicians and patients with regards to treatment decisions and adjuvant treatment trial design.
